# A draft genome assembly of the reef-building coral *Acropora hemprichii* from the central Red Sea

**DOI:** 10.1038/s41597-024-04080-8

**Published:** 2024-11-26

**Authors:** Anna Fiesinger, Carol Buitrago-López, Abdoallah Sharaf, Anny Cárdenas, Christian R. Voolstra

**Affiliations:** 1 Department of Biology, University of Konstanz, Konstanz, Germany; 2 General Organization for the Conservation of Coral Reefs and Turtles in the Red Sea (Shams), Jeddah, Saudi Arabia; 3 SequAna Core Facility, Department of Biology, University of Konstanz, Konstanz, Germany; 4 Department of Biology, American University, Washington, DC 20016 USA

**Keywords:** Molecular ecology, Molecular evolution

## Abstract

Coral reef ecosystems are under threat from climate change. Thus, active interventions to spur coral conservation/restoration are critical to support reef survival, greatly informed by a molecular understanding of resilience. The genus *Acropora* is a species-rich and globally prevalent reef builder that has experienced dramatic declines in the Caribbean. Here we generated a draft genome of the common coral *Acropora hemprichii* from the central Red Sea, one of the warmest water bodies in the world. We assembled the genome using 10x Chromium sequencing with subsequent scaffolding using a reference genome and Illumina short-read sequencing contigs. The *A. hemprichii* genome has an assembly size of 495.6 Mb confirmed using physical size estimation, of which 247.8 Mb (50%) are repeats. The scaffold N50 is 1.38 Mb with 99.6% of BUSCO genes identified (93.7% complete, 5.9% fragmented), providing a set of 26,865 protein-coding genes. The Red Sea *A. hemprichii* reference genome provides a valuable resource for studies aiming to decode the genomic architecture of resilience, e.g. through comparative analyses with other *Acropora* genomes.

## Background & Summary

Coral reefs support approximately one-third of the world’s multicellular marine biodiversity^1^ and close to one billion people^2^. Coral reefs are one of the ecosystems most affected by climate change^3,4^, and reef-building corals have been experiencing widespread mortality over recent decades due to local and global anthropogenic impacts^5–7^. Climate change is one of the main drivers of reef decline as ocean warming causes extended heat stress episodes that in turn trigger coral bleaching, i.e. the loss of dinoflagellate symbionts of the family Symbiodiniaceae^6,8,9^. As a consequence, corals eventually die, as the highly efficient nutrient cycling between corals and their endosymbiotic photosymbionts is impaired^9,10^. It is currently unclear to what extent or at what rate corals can adapt to the increasing frequency and extent of thermal stress events^11–13^. Increasing the availability of coral genomes, particularly from warm water bodies, is an important step to providing resources that facilitate decoding the genomic architecture of thermal resilience.

The coral genus *Acropora* is a widely distributed, speciose genus, comprising over 400 nominal and more than 100 described species^14,15^. *Acropora* corals provide habitat and shelter for an extensive array of marine species due to their complex branching growth forms^16^. Their fast-growing nature makes them a primary target for restoration efforts, since they are easy to fragment and accumulate biomass quickly^17,18^. They are, however, highly vulnerable to environmental stress and coral bleaching, with more than 70% of acroporid species currently classified as near threatened or threatened on the International Union for Conservation of Nature Red List^19^.

**Fig. 1:**
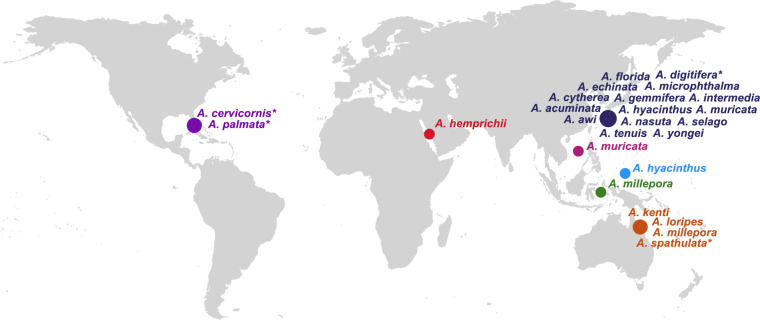
Overview of publicly available *Acropora* genomes with sampling locations of each species indicated by dots. *Several genomes from multiple specimens exist for *A. cervicornis*, *A. digitifera*, *A. palmata*, and *A. spathulata* from the same geographic region. Please refer to Table S1 for further details on each genome.

Given the ecological (and economic) importance of *Acropora* species, the sequencing of the *Acropora digitifera* genome from Okinawa, Japan^20^ marked a milestone as the first coral genome to be fully sequenced. A growing body of additional coral genomes has since become available^21–26^, with the genus *Acropora* having by far the most sequenced genomes. These genomes are from various geographic origins and feature a range of sequencing and assembly approaches including several chromosome-scale assemblies such as *Acropora millepora*^24^, *Acropora palmata*^27^, *Acropora hyacinthus*^28^, and *Acropora cervicornis*^29^. However, to date no *Acropora* genome has been published from the Red Sea. The here-provided draft genome will thus further contribute to the broad taxonomic and spatial coverage of available *Acropora* genomes (Fig. 1, Table S1). Deciphering genomes of species from high-temperature marine environments is crucial, as they can provide insight with regard to the genomic architecture and genes underlying thermal resilience. Red Sea corals have been shown to be exceptionally thermally resilient, especially those from the Northern Red Sea^30–33^. Transcriptomic resilience and the constitutive expression (‘front-loading’) of thermal stress genes were identified as two of the putative underlying mechanisms^33,34^. *Acropora* species are still abundantly present throughout the Red Sea but are considered extinct in the Persian/Arabian Gulf, due to ongoing urbanization and severe marine heat waves in recent decades^35^.

**Fig. 2:**
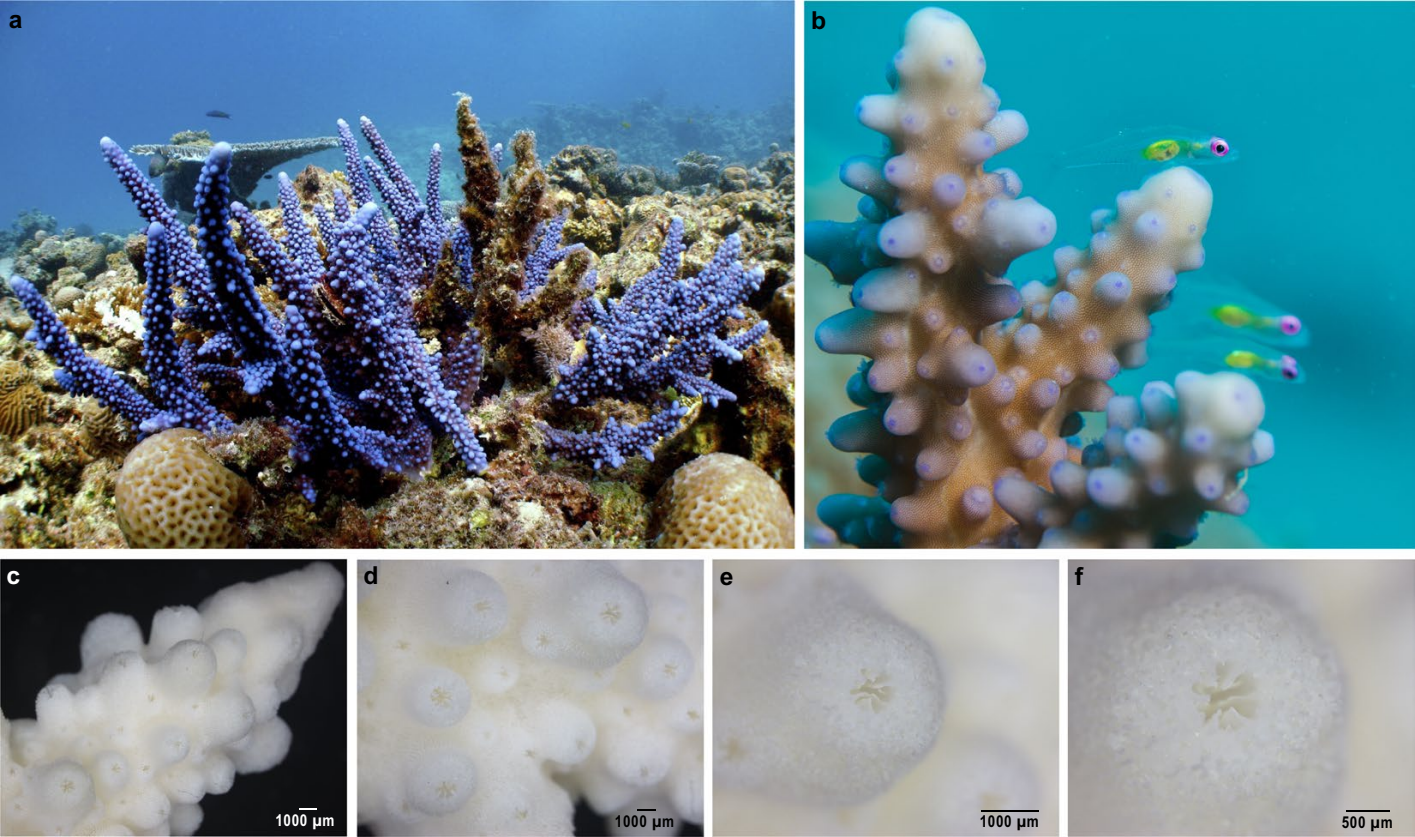
*Acropora hemprichii* from the Red Sea. **(a)** *in situ* colony; **(b)** *in situ* closeup of three nubbins; **(c - f)** Stereomicroscope images of *Acropora hemprichii* skeleton, whereby **(e, f)** show a closeup of corallites. Coral skeleton images were taken with a Nikon SMZ 25 Optical Stereo Microscope using the NIS-Elements software. Note that the colonies depicted here do not correspond to the colonies sampled for DNA and RNA sequencing. Taxonomic verification of sampled specimens was conducted by Dr. Francesca Benzoni and Dr. Tullia Terraneo at King Abdullah University of Science and Technology (KAUST). Picture credits: (a) Michael L. Berumen; (b) Anna Roik; (c - f) Gustavo Ramirez-Calderon.

To facilitate genomic comparisons underlying thermal resilience, we generated a draft genome using short- and linked-reads of the stony coral *Acropora hemprichii* with an assembly size of 495.6 Mb, a scaffold N50 of 1.38 Mb, and 99.6% of BUSCO genes identified (93.7% complete, 5.9% fragmented). This is the first draft genome assembly of an *Acropora* species from the Red Sea, one of the warmest regions in the world. *Acropora hemprichii* (Ehrenberg, 1834) is a significant reef-builder in the Red Sea where it dominates shallow reef slopes^36^. *A. hemprichii* is endemic to the Red Sea and the Indian Ocean^37^. With its blue-ish color (Fig. 2), it is easy to distinguish in the reef, which may explain the numerous physiological studies available^32,38,39^. *A. hemprichii* has been shown to associate with diverse bacterial communities that rapidly adjust under changing environments, which may constitute one of the reasons explaining its prevalence across the adverse environmental gradients of the Red Sea^40,41^. The high contig and scaffold N50s of this draft assembly should allow for interesting evolutionary comparisons with other available coral genomes.

## Methods

### Sample collection and nucleic acid isolation

A colony of *Acropora hemprichii* (Fig. 2) was collected from Abu Shosha (22°18′16.8″N 39°02′56.8″E), a nearshore reef in the central Red Sea, on March 2nd, 2019. The colony was then transferred to the aquaria facilities of the Coastal and Marine Resources Core Lab (CMOR) at KAUST, Saudi Arabia, where it was reared using a seawater flow-through system. Approximately one month later, a fresh 5 cm nubbin was collected from the colony and preserved in DESS buffer overnight at 4 °C^42,43^. The coral tissue was then air-blasted from the skeleton using a cold, filtered seawater NAC (2%) mucolytic solution. The resultant coral slurry was homogenized and centrifuged to enrich the coral host fraction for DNA extraction and nuclei isolation, following procedures described in a previous study^23^.

### Genome sequencing

High molecular weight genomic DNA (HMW gDNA) was extracted using the Qiagen genomic tip 100/g kit following the manufacturer’s instructions. HMW gDNA integrity and quality were assessed using 1% agarose gel electrophoresis and a NanoDrop2000 spectrophotometer (Thermo Fisher Scientific, Waltham, Massachusetts, USA). The gDNA concentration was determined using a Qubit dsDNA Broad Range assay (Thermo Fisher Scientific, Waltham, Massachusetts, USA). A total of 20 µg DNA were submitted to the KAUST Bioscience Core Lab (BCL) for the construction of a 10x Chromium sequencing library following the manufacturer’s instructions, which was sequenced on a HiSeq4000 lane (2 × 151 bp) (Table 1). In addition, a paired-end library was constructed using the NEBNext Ultra II DNA kit following the manufacturer’s instructions. Briefly, 1 µg DNA was fragmented by Adaptive Focused Acoustic Technology (Covaris, Woburn, Massachusetts, USA) to a target size of 300 bp to achieve overlapping paired-end reads, a requirement for the DISCOVAR *de novo* genome assembler (see below). The fragmented DNA was end-repaired and dA-tailed for Illumina adapter ligation. Adapter-ligated DNA was purified and size selected for fragments within a 400 bp range using AMPURE XP magnetic beads (Beckman Coulter, Brea, California, USA). The resulting product was subjected to PCR amplification using the NEBNext primer cocktail and Q5 Master Mix. To ensure an adequate quantity of library fragments for sequencing while minimizing PCR-related artifacts, a conservative approach was taken with only five PCR cycles for library amplification. The final library was quality-checked by assessing both its size (~400 bp) using a 2100 Bioanalyzer (Agilent, Santa Clara, California, USA) and its concentration using a Qubit fluorometer and the Qubit High Sensitivity dsDNA assay kit (Thermo Fisher Scientific, Waltham, Massachusetts, USA). The paired-end library was sequenced at Macrogen on one Illumina HiSeq2500 lane (rapid run 2 × 251 bp) (Table 1).

**Table 1:** Summary of gDNA and RNA sequencing data for *Acropora hemprichii*.

Sample	Library type	Sequencing platform	Raw data (no. of reads)	Filtered data (no. of reads)
**Genome**				
Chromium reads	10x Chromium	HiSeq4000 (2 × 151 bp)	307,601,772	307,601,772*
Short reads (overlapping PE)	Shotgun	HiSeq2500 (2 × 251 bp)	249,281,840	238,221,412
**RNA-Seq**				
Ahem	Strand-specific mRNA	HiSeq4000 (2 × 151 bp)	253,314,988	152,357,046
HE1-33	Strand-specific mRNA	HiSeq4000 (2 × 151 bp)	236,556,750	173,008,312
HE1-36	Strand-specific mRNA	HiSeq4000 (2 × 151 bp)	226,215,752	152,357,046

*Following the Supernova assembly process documentation, the standard assembler for 10x Chromium reads, barcoded Chromium reads were not filtered before assembly.

### RNA sequencing

RNA was isolated from the same colony that was used for genome sequencing (Table 1). In addition, to increase the diversity of expressed transcripts, we extracted RNA from two *Acropora hemprichii* nubbins of a different colony exposed to either low (33 °C) or high (36 °C) heat stress (3 h ramp-up to heat stress, 3 h heat-hold at respective heat stress target temperatures) using the Coral Bleaching Automated Stress System (CBASS)^44,45^ (Table 1). Nubbins were collected after an overnight recovery (11 h) phase in the dark. Upon collection, fragments were flash-frozen in liquid nitrogen and stored at −80 °C. For all RNA extractions, frozen *A. hemprichii* nubbins were placed in a sterile bag and the coral tissue was air blasted off the skeleton into 1 mL sterile artificial seawater. The resulting slurries were homogenized using a tissue homogenizer. A total of 200 μL of the homogenate were mixed with 200 μL RNeasy lysis buffer (Qiagen), snap-frozen in liquid nitrogen, and stored at −80 °C. Total RNA was extracted using the RNeasy Mini Kit (Qiagen) following the manufacturer’s protocol. RNA quality was assessed on a 2100 Bioanalyzer (Agilent Technologies). Libraries for all three samples were prepared from ~1 μg total RNA using the TruSeq Stranded mRNA kit (Illumina, San Diego, CA, USA) following manufacturer’s instructions. The SuperScript II Reverse Transcriptase kit was used for the cDNA synthesis step and Agencourt AMPure XP beads for cleanup steps according to the manufacturer guidelines. Final libraries (average insert size ~250 bp to generate overlapping paired-end reads) were quality-checked on a 2100 Bioanalyzer using the High Sensitivity DNA assay and sequenced in paired-end mode (2 × 151 bp) on a HiSeq4000 at the KAUST BCL. The quality of RNA-Seq reads was checked using FastQC^46^ to determine that reads were of sufficient quality for transcript mapping.

### Estimation of genome size

**Fig. 3:**
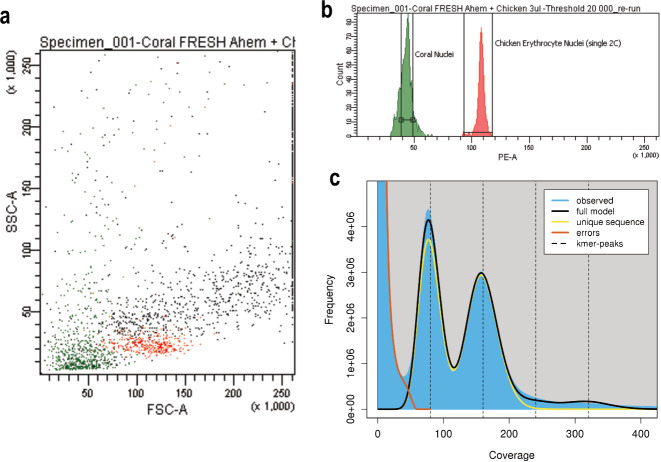
Genome Size Estimation. (**a)** Flow cytometry-based estimation showing the distribution of nuclei populations based on light scatter in the Forward (FSC) and Side (SSC) Scatter Channels, with intact coral and chicken single nuclei represented in green and red, respectively. **(b)** Fluorescence intensity histogram of *A. hemprichii* (green) and chicken erythrocyte nuclei (red). **(c)** Genome size estimation plot based on k-mer (k = 21) distribution using jellyfish v2.2.10 and GenomeScope 2.0. The analysis reveals a genome size of 428.1 Mb that is largely unique (57.9%), predominantly homozygous (98.3% AA), and with very little heterozygosity (1.7% AB).

The physical genome size of *A. hemprichii* was estimated by nuclei size estimation in reference to chicken erythrocyte nuclei as a standard (DNA QC Particles kit, BD Biosciences, San Jose, CA), following a previous protocol of propidium iodide stained nuclei on the LSRII Fortessa (BD Biosciences, San Jose, CA, USA) using a 561 nm laser and a BP610 ± 20 nm filter^23^. Estimation of the coral genome size was in reference to the diploid DNA content of chicken erythrocytes, measured at 2.5 pg ± 0.04 per cell. Determination of the haploid genome size [pg] was calculated using the equation: 1.25 * x/y (where x represents the fluorescence intensity of the unknown sample and y represents the fluorescence intensity of chicken erythrocyte nuclei). The mean DNA content per haploid genome or copy of genetic information (1 C) was calculated as ~498 Mb, considering the equivalence of 1 pg DNA to 978 Mb^47^ (Fig. 3a,b). The genome size was also bioinformatically estimated using a k-mer histogram generated with jellyfish v2.2.10 based on the 10x Chromium and Illumina sequencing reads and visualized in GenomeScope 2.0^48^. This approach provided a genome size estimate of 428.1 Mb with a heterozygosity of 1.74% at a k-mer size of 21 (Fig. 3c).

### Genome assembly and repeat annotation

**Fig. 4:**
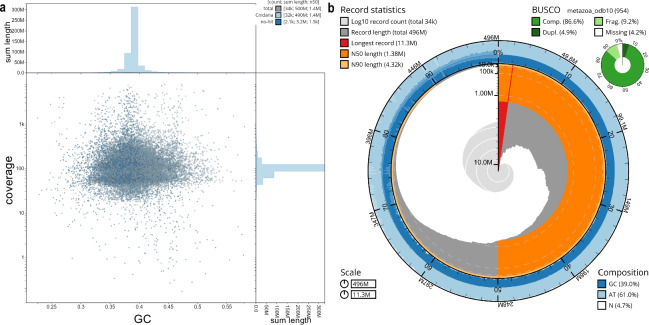
Quality assessment of the *Acropora hemprichii* genome assembly using BlobToolKit v2. (**a)** Blob plot showing absence of putative contamination by non-target species (after filtering); **(b)** Snail plot showing contig assembly and GC distribution, as well as BUSCO completeness (based on BUSCO metazoa_odb10, Table 2).

*De novo* assembly of short- and linked-reads generated from 10x Chromium libraries (henceforth referred to as Chromium reads) was performed using Supernova v2.1.1^49^ using default settings. Reference scaffolding of the raw assembly was done with RagTag v1.1.1^50^ using the highly complete genome of *Acropora tenuis*^51^ as a reference. Gap filling of the scaffolded genome assembly was done using LR_Gapcloser v3^52^ with contigs generated from a *de novo* assembly using DISCOVAR^53^, based on the trimmed and filtered overlapping paired-end reads using trimmomatic v0.39^54^ following previously described procedures^23^. The quality of the assembled genome was assessed using BlobToolKit (BTK) v2^55^, prompting the removal of 3,487 contigs due to short length, low coverage, and/or similarity to non-related taxa (Fig. 4). This yielded 33,983 scaffolds with a total length of 495.6 Mb and a scaffold N50 of 1.38 Mb (Table 2). This assembly size is very close to the expected genome size obtained from nuclei size estimation. DNA repeats in the genome were identified using a combination of RepeatModeler v2.0.4^56^ and the EDTA pipeline^57^, and masked using RepeatMasker v4.1.6^58^ with custom *Acropora*-specific repeat libraries under the “LTRStruct” option and the default setting for all other parameters. Repetitive sequences comprised a total of 247,816,887 bp (50.00%) of the assembled genome, including transposable elements (22.29%) and unclassified repeats (27.32%) (Table S2).

**Table 2:** Genome assembly statistics of *Acropora hemprichii*.

General statistics	
Estimated genome size (physical; Mb)	498
Estimated genome size (k-mer; Mb)	428.1
Assembled genome size (Mb)	495.6
Number of scaffolds	33,983
Scaffold N50 (Mb)	1.38
Longest scaffold (Mb)	11.3
Shortest scaffold (bp)	818
Average scaffold length (Kb)	14,568
Number of scaffolds >50 Kb	517
Number of contigs	55,122
Contig N50 (Kb)	23
Longest contig (Kb)	226
Shortest contig (bp)	48
Average contig length (Kb)	8.5
Repeat content (%)	50
GC content (%)	38.97
Number of gaps in scaffolds	21,205
Total gap length in scaffolds (bp)	23,378,281
Average gap length in scaffolds (bp)	1,102
**BUSCO (eukaryota_odb10)**	
Complete (%)	93.7
- Single-copy (%)	88.6
- Duplicated (%)	5.1
Fragmented (%)	5.9
Missing (%)	0.4
**BUSCO (metazoa_odb10)**	
Complete (%)	89.9
- Single-copy (%)	84.8
- Duplicated (%)	5.1
Fragmented (%)	6.6
Missing (%)	3.5

### Gene prediction and annotation

Gene prediction was performed using funannotate v1.8.16^59^. First, RNA-Seq reads were mapped to the assembled genome using STAR v2.7.10^60^ and transcripts were derived from the alignment using StringTie v2.2.0^61^. Open reading frames (ORFs) were retrieved using TransDecoder v5.7.1^62^. Second, *Acropora* proteins were retrieved from UniProt (SwissProt and TrEMBL; date of access: 29.01.2024) and used as protein evidence in the funannotate pipeline^63^. *Ab initio* gene predictors used in the funannotate pipeline are GeneMark-ES^64^, AUGUSTUS^65^, HiQ (high-quality AUGUSTUS), CodingQuarry^66^, GlimmerHMM^67^, and SNAP^68^. CodingQuarry uses the RNA-Seq mapping information as training data, whereas all other predictors use the identified BUSCO gene set as a training file. Finally, using the EVidenceModeler (EVM) software^69^, all *ab initio* gene predictions as well as protein-translated and transcript alignments were combined into weighted consensus gene structures. Gene models that were shorter than 100 amino acids or consisted of transposable elements were removed using DIAMOND v2.1.8 BLASTp^70^. Bedtools intersect v2.31.0^71^ was used to find overlaps between predicted genes and identified repeats (parameters: -sorted -f 0.9). The longest isoform was kept for each predicted gene using the script agat_sp_keep_longest_isoform.pl implemented in AGAT^72^, yielding a total of 26,865 predicted protein-coding gene models (Table 3, Table S3). In addition, 243 high-confidence transfer RNAs were predicted using tRNAscan-SE v2.0.12 based on filtering of the initial set of 6,810 putative tRNAs using EukHighConfidenceFilter^73^ (Table S4).

**Table 3:** Genome annotation statistics for *Acropora hemprichii*.

Structural annotation	
Protein-coding genes	26,865
High confidence tRNAs	243
Average gene length (bp)	4974.12
Average protein length (bp)	441.06
**Proteins with available functional annotation**	
Total number of protein-coding genes	26,865
eggNOG	23,541 (87.63%)
COG	21,943 (81.68%)
InterProScan	21,547 (80.20%)
Pfam	17,706 (65.91%)
GO terms	16,085 (59.87%)
Phobius	9,222 (34.33%)
UniProt	1,683 (6.26%)
MEROPS	1,009 (3.76%)
BUSCO	983 (3.67%)
CAZYme	458 (1.71%)
**BUSCO (eukaryota_odb10)**	
Complete (%)	83.9
- Single-copy (%)	79.2
- Duplicated (%)	4.7
Fragmented (%)	12.5
Missing (%)	3.6
**BUSCO (metazoa_odb10)**	
Complete (%)	83.0
- Single-copy (%)	78.0
- Duplicated (%)	5.0
Fragmented (%)	9.4
Missing (%)	7.6

The predicted genes were annotated using a combination of databases and tools. We used InterProScan v5.65-97.0^74^, eggNOG-mapper v2^75^, BUSCO^76^, and COGs (Clusters of Orthologous Groups of Proteins)^77^ for the functional annotation. Further, CAZy (carbohydrate-active enzymes database)^78^ was used for the annotation of carbohydrate-active enzyme families, MEROPS^79^ for the classification of proteases, and Pfam^80^, UniProt, and Phobius^81^ were queried for transmembrane topology and signal peptide prediction. The list of available annotations for the set of 26,865 genes is available in the supplement (Table S5).

### Comparative genomics analyses

**Fig. 5:**
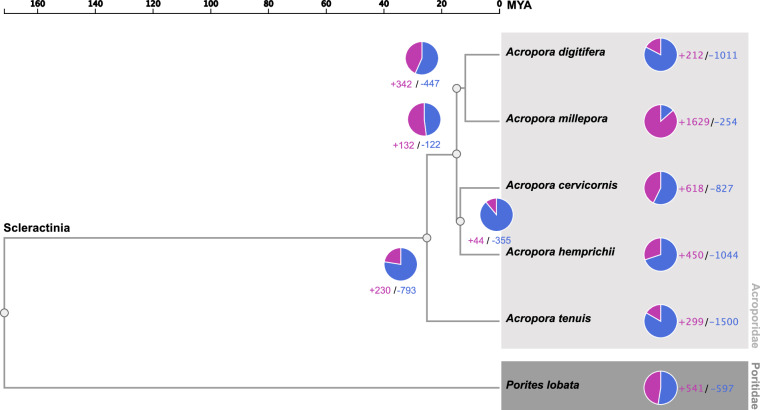
Maximum likelihood (ML) phylogenetic tree based on single-copy ortholog groups (i.e., 1-to-1 orthologs) across *A. digitifera*, *A. millepora*, *A. cervicornis*, *A. hemprichii* (this study), *A. tenuis*, and *Porites lobata* (outgroup). The inferred divergence time is based on the TIMETREE5 database (with an estimated median divergence time between Acroporidae and Poritidae of ~172 MYA). The tree was constructed with the evolution model ‘JTT + CAT’ in FastTree v2.1.11 within the OrthoVenn3 pipeline. Ortholog group (OG) expansions are denoted in purple (+), OG contractions are denoted in blue (−), calculated with CAFÉ5 for each node. Bootstrap support is 100 for each node.

Ortholog gene clusters among *A. hemprichii* (this genome), *A. cervicornis*^82^*, A. digitifera*^26^, *A. tenuis*^26^*, A. millepora*^24^, and *Porites lobata*^83^ as the outgroup were identified using OrthoVenn3^84^ utilizing the OrthoFinder algorithm^85^. To do this we used the predicted protein sequences of the genome either from provided fasta files or extracted from gff files. This analysis identified 3,608 single-copy orthologs (1-to-1 orthologs) across all species^21^. Orthologs were first individually aligned across species using MUSCLE v5.1^86^, then trimmed using trimAl v1.4^87^, and finally concatenated to construct a maximum-likelihood (ML) tree based on the evolution model ‘JTT + CAT’, which was inferred using FastTree v2.1.11^88^. Inferred divergence times were based on the TimeTree5 database^89^, using a median divergence time of Acroporidae vs. Poritidae of ~172 MYA based on four studies that detail molecular data and/or fossil records. The ‘SH test’ method was used to assess the credibility of each phylogenetic node. Results from the ortholog gene cluster analysis (Table S6) were mapped onto the phylogenetic tree to assess gene family expansion and contraction for each node using CAFÉ v5.1^90^ and a *p*-value cutoff of <0.05. This analysis confirmed that *A. hemprichii* is the sister species of *A. cervicornis* with 450 expanded and 1044 contracted ortholog gene clusters (OGs) in *A. hemprichii* (Fig. 5; Table S7). Further, we identified 380 OGs that were only present in *A. hemprichii* and absent in the other 5 species (Fig. S1). This set of 380 OGs comprised a total of 892 genes, representing species-specific genes assumed to provide insight in the unique evolutionary adaptations^91,92^. Gene Ontology (GO)-based functional enrichment^93^ of this species-specific gene set was assessed against the background of 10,459 OGs shared among all six species with a *p*-value cutoff of <0.05 (Fig. S2). The 380 OGs were enriched for a range of biological processes and cellular components, including membrane functioning (GO:0016020), viral processes (GO:0016032), RNA splicing (GO:0008380), and response to oxidative stress (GO:0006979) (Table S8). The *A. hemprichii* OGs were majoritively composed of two to four orthologs, while three OGs comprised eight genes: Two of these OGs were orthologs without annotations and one OG with genes annotated as Far upstream element-binding protein 3 (FUBP3) that belongs to a family of single-strand DNA-binding transactivators, putatively involved in splicing repression^94,95^. Comparing the presence, structure, and sequence conservation of orthologous genes in the here assembled genome against well-annotated genomes, provides an indication of completeness and accuracy of the assembly. The phylogenetic placing based on the orthologs analysis further supports the high quality of the draft genome assembly.

## Data Records

The dataset is available at NCBI SRA under SRP507249^96^, at NCBI GenBank under JAZHQP000000000.1^97^, and under the BioProject PRJNA1027610. The BioProject contains information on and links to samples, unprocessed (raw) sequencing fastq files (DNA and RNA), and the genome assembly (GenBank Accession Number: JAZHQP000000000). The genome annotation file (Acropora_hemprichii.gff3.gz), predicted genes files (Acropora_hemprichii_final_CDS_26865.fa.gz, Acropora_hemprichii_final_aa_26865.fa.gz), and the annotation file (Acropora_hemprichii_final_annot_protein-coding_26865.txt.gz) are available at http://ahem.reefgenomics.org^98^ and at the corresponding Zenodo repository at 10.5281/zenodo.13907127^99^.

## Technical Validation

The quality of the assembled *A. hemprichii* genome was assessed using several approaches: (i) gDNA integrity and quality were inspected using 1% agarose gel electrophoresis and a NanoDrop2000 spectrophotometer, which indicated pure, high molecular weight DNA in the form of a distinct band above the 10 kb marker in the gel as well as 230/260 and 260/280 spectrophotometric readings >1.8; the gDNA concentration was determined using a Qubit dsDNA Broad Range assay; (ii) the estimated genome size was compared using a physical genome size estimation approach yielding a total size of ~498 Mb with the estimated genome size using a kmer-based approach yielding a total size of 428.1 Mb (Fig. 3) and a total assembly size of 495.6 Mb; (iii) contamination of the assembly was assessed to be low using BlobToolKit (Fig. 4a) after removal of contigs that did not map to coral, with a GC content of 38.97% and a scaffold N50 of 1.38 Mb; (iv) genome completeness was further assessed using BUSCO (Fig. 4b) with 93.7% complete genes (88.6% single-copy, 5.1% duplicated) and a further 5.9% fragmented genes, totaling 99.6% (eukaryota_odb10), thus suggesting that the sequencing and assembly procedure has reconstructed a reliable representation of the full set of genes; (v) gene completeness was assessed using BUSCO with 83.9% complete (79.2% single-copy, 4.7% duplicated) and a further 12.5% fragmented genes, totaling 96.4% (eukaryota-odb10). The predicted gene set was assessed further for completeness and potential contamination using OMArk^100^, which revealed 92.14% complete eumetazoan hierarchical ortholog groups (HOGs) and no contamination (Fig. S3). These results indicate that the *A. hemprichii* assembly is of high quality.

## Supplementary information

Supplementary Material Figures

Supplementary Material Tables

## Data Availability

All bioinformatic tools used to assemble and annotate the *A. hemprichii* genome are referenced here: https://github.com/Carol-Symbiomics/Acropora-hemprichii-genome-assembly. The version and parameters used are described below. Parameters are default if not specified otherwise. (1) FastQC v0.12.1 (2) Trimmomatic v0.39 (3) BBmap v38.76 (4) DISCOVAR (5) Supernova v2.1.1 (6) BUSCO v5.7.1, parameters: lineage_dataset eukaryota_odb10 (255 BUSCOs) and metazoa_odb10 (954 BUSCOs) (7) BlobtoolKit v2 (8) Jellyfish v2.2.10 (9) GenomeScope v2.0: parameters: k-mer 21 ploidy 2 (10) Smudgeplot v0.2.5 (11) RepeatModeler v2.0.4 (12) EDTA v2.2.0 (13) RepeatMasker v4.1.6, option “LTRStruct” (14) STAR v2.7.10 (15) StringTie v2.2.0 (16) TransDecoder v5.7.1 (17) Funannotate v1.8.16 (a) predict, parameters: -s “Acropora hemprichii” –organism other –buco_db metazoa –min_protlen 100; (b) fix (c) annotate, parameters: -s “Acropora hemprichii” –busco_db metazoa (18) GeneMark-ES, parameters:–max_intron 3000–soft_mask 2000 (19) AUGUSTUS v3.5.0 (20) CodingQuarry v2.0 (21) GlimmerHMM v3.0.4 (22) SNAP (23) Exonerate v2.4.0 (24) DIAMOND v2.1.8, parameters: blastp –sensitive –evalue 1e-10 –max-target-seqs. 1 –outfmt 5 (25) Tbl2asn v25.8 (26) Bedtools v.2.31.0, parameters: intersect -sorted -v (27) Bedtools v.2.31.0, parameters: intersect -sorted -f 0.9 (28) Bam2hints (29) tRNAscan-SE v2.0.12 (30) AGAT (Another Gtf/Gff Analysis Toolkit) (31) Minimap2 v2.26-r117, parameters: -ax splice–cs -u b -G 3000 (32) Phobius v1.01, parameters: -short (33) eggNOG-mapper v2.1.11, parameters: -m diamond (34) InterProScan v5.65-97.0, parameters: -t p -goterms -pa (35) MEROPS v12.0 (36) Pfam v35.0 (37) CAZYmes v12.0 (38) OMArk v0.3.0, NCBI taxid: 213635 (39) OrthoVenn3, parameters: e-value 1e-5, inflation value 1.5 (40) OrthoFinder (41) MUSCLE v5.1 (42) trimAl v1.4 (43) FastTree v2.1.11 (44) TIMETREE5 (45) CAFÉ v5.1 (46) RStudio v2023.06.0
