# Supplementary material for: A draft genome assembly of the reef-building coral Acropora hemprichii from the central Red Sea
Source: Sci Data. 2024 Nov 26;11:1288. doi: 10.1038/s41597-024-04080-8 (PMC11599867; doi:10.1038/s41597-024-04080-8)
Supplement: Supplementary file 1 — Supplementary Material Figures [file 41597_2024_4080_MOESM1_ESM.docx]

**Supplementary Material**

**Supplementary Figures**


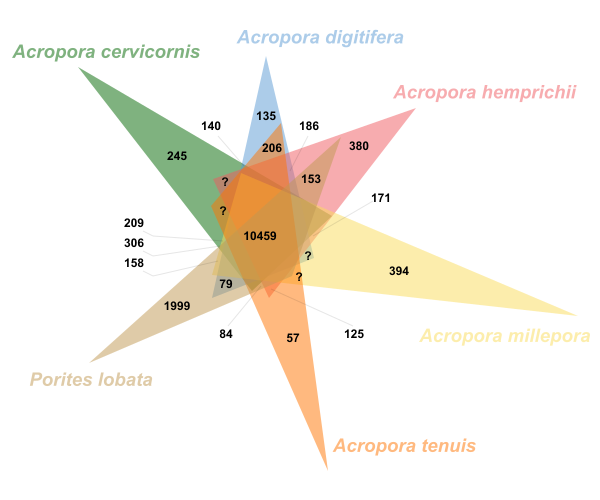


**Figure S1.** Venn diagram of shared and specifc ortholog groups between *A. cervicornis*, *A. digitifera*, *A. tenuis*, *A. millepora*, *A. hemprichii*, and *Porites lobata*.


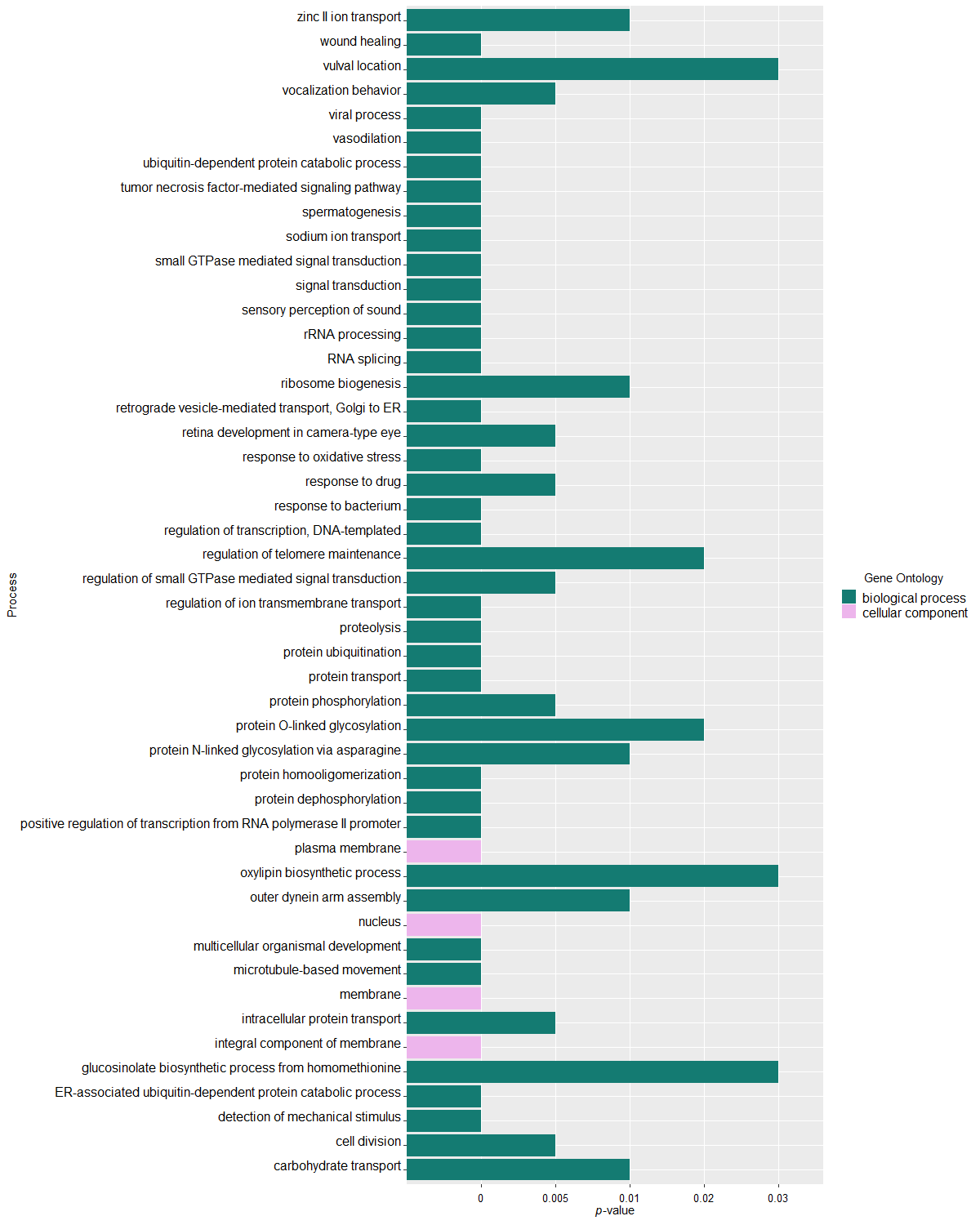


**Figure S2.** Gene Ontology (GO) functional enrichment analysis (*p*-value < 0.05) for 380 *A. hemprichii*-specific ortholog groups based on OrthoFinder as implemented in OrthoVenn3. The plot was generated using ggplot2 in RStudio v2023.06.0.


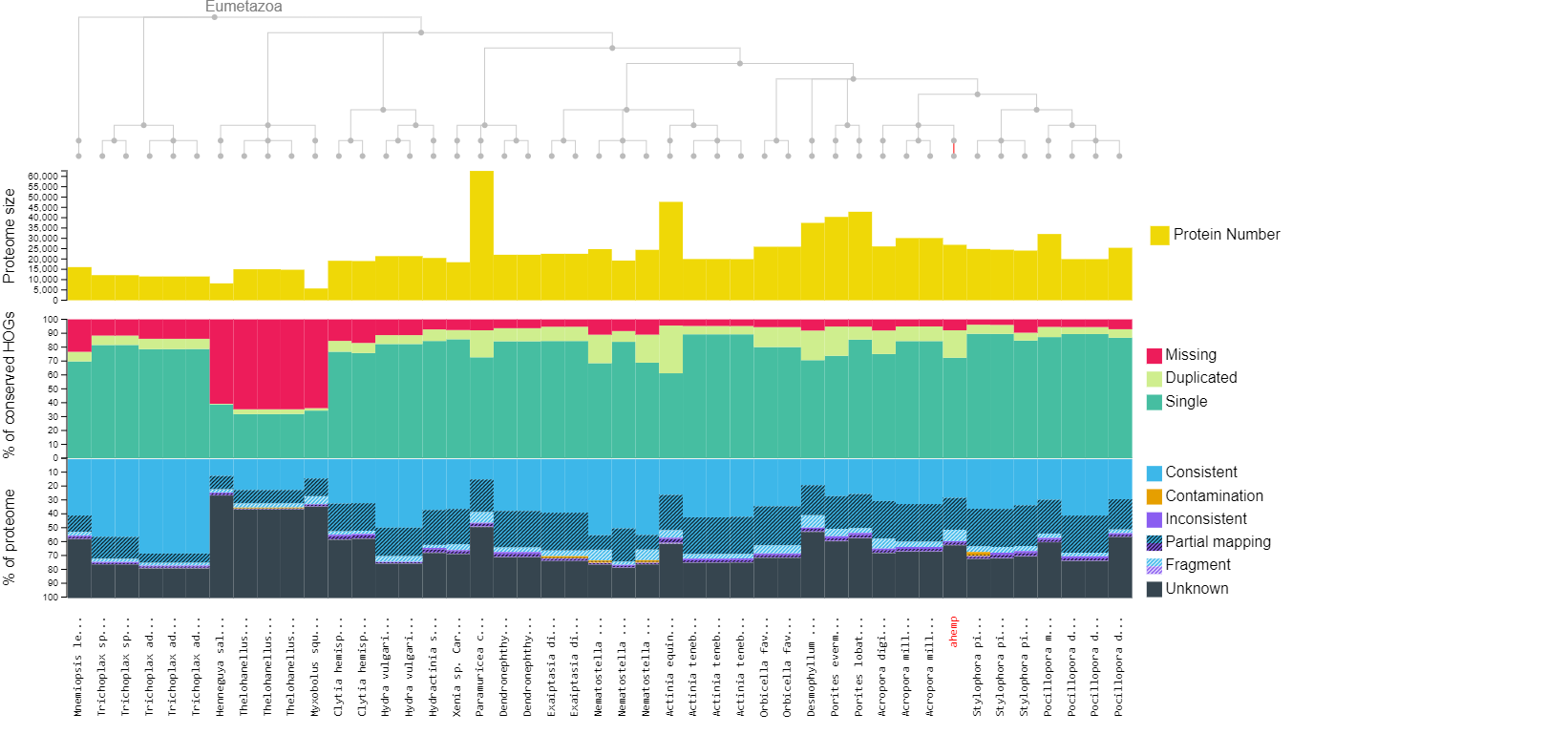


**Figure S3.** Estimation of the predicted gene set completeness of *Acropora hemprichii* (ahemp; red) in comparison to other available coral genomes, based on presence/absence of conserved orthologs groups within the Eumetazoa (2,355 single-copy orthologs) using the OMArk webinterface (<https://omark.omabrowser.org/>).
